# Early Diagnosis of Lung Cancer: The Urgent Need of a Clinical Test

**DOI:** 10.3390/jcm11154398

**Published:** 2022-07-28

**Authors:** Roberto Gasparri, Alessandra Guaglio, Lorenzo Spaggiari

**Affiliations:** 1 Department of Thoracic Surgery, IEO, European Institute of Oncology IRCCS, 20141 Milan, Italy; alessandra.guaglio@ieo.it (A.G.); lorenzo.spaggiari@ieo.it (L.S.); 2 Department of Oncology and Hemato-Oncology, University of Milan, 20122 Milan, Italy

Globally, lung cancer continues to be the leading cause of cancer death in men and women [1,2]. In fact, it is estimated that more people die each year from lung cancer than from colon, breast, and prostate cancer combined [3]. The main cause of these dramatic epidemiological data is the fact that there are still no adequate and effective screening methods. In fact, to date, only 15% of patients are diagnosed at an early stage [4]. On the other hand, mammography [5], fecal occult blood detection [6], and PSA screening [7] provide better results in terms of both long-term survival and cancer mortality.

Nowadays, the annual screening of high-risk populations with low-dose computed tomography (LDCT) is both recommended and clinically approved by the recently published NCCN 2021 guidelines for lung cancer screening [8]. However, its effectiveness in increasing survival rates is still debated, mainly because the criteria for defining high-risk groups have not yet been clearly defined [9]. In addition, the high false-positive rate, costs, and the exposure to potentially harmful radiation may limit its use in the clinical routine. In particular, overdiagnosis leading to unnecessary interventions has important effects on both health systems and the psychology of patients [10]. From a clinical point of view, most lung cancer patients are diagnosed in advanced stages or metastatic disease, overburdening the healthcare system to access combined chemo-/radiotherapy treatments, despite their poor survival and mortality rates. On the opposite, surgical treatment for resectable and operable early stage disease can not only be curative, but provides five-year survival rates after surgical resection of 30–80% compared to 2–15% for advanced or metastatic stages [11]. It is clear, therefore, that the development of better screening tools enabling early diagnosis is the key factor in improving the prognosis of lung cancer patients.

Advances in the early diagnosis of lung cancer have increased during the last decades, especially for the “OMIC” approaches such as volatolomic, genomic, transcriptomic, proteomic, and metabolomic [12,13,14]. These techniques help to understand cancer at different levels of complexity and as an exogenous system inside the host body. Several technologies have been developed and a number of predictive biomarkers have been investigated in many platforms with promising results. Although promising, no predictive biomarkers of disease onset—indicators of the disease at an early stage before becoming symptomatic or detectable by conventional means—have proven to be clinically useful [15]. Moreover, one of the major challenges that are linked to these approaches is the large heterogeneous amount of data generated from the analysis of a single patient, which need to be translated into clinically useful information [16,17]. In this context, artificial-intelligence (AI) and machine-learning (ML) frameworks, which summarize and correlate information from several sources and provide accurate predictions, are a powerful, innovative, and potentially very efficient instrument [18]. 

Now is the time to focus on the prospective research for a panel of diagnostic biomarkers that can be used in the clinical setting. It is crucial to merge the work of clinicians with that of researchers to develop and validate a diagnostic tool that uses AI\ML to integrate patients’ data about tumor-specific biomarkers, identified by analyzing accessible biological samples, such as urine, blood, and exhaled breath. The costs of such analyses must be limited in order to make them reproducible and affordable even for less wealthy countries. All the researchers performing trials focusing on biomarkers for the early detection of lung cancer must set up a task force in their own country and interface with other countries with the aim of sharing results as well as exchanging knowledge. For this aim, it is important to create an Omics Big Data Bank in which all patients’ data could be recorded and analyzed sequentially, even many years after the first collection. This set of predictive data could be used as a guide by the clinicians in the decision of the follow-up program of the patient and to orientate them in the therapeutic decision. Acting as soon as possible could significantly improve disease-related quality of life and survival. 
